# Hématome sous dural fœtal

**DOI:** 10.11604/pamj.2013.15.17.2512

**Published:** 2013-05-08

**Authors:** Hanane Saadi, Abdelaziz Banani

**Affiliations:** 1 Service de gynécologie obstétrique I CHU Hassan II, Fès, Maroc

**Keywords:** Hématome sous dural fœtal, diagnostic anté natal, pronostic, Fetal subdural hematoma, ante-natal diagnosis, prognosis

## Image en médicine

L'hématome sous dural in utero (HSD) est très rare, se soldant fréquemment par le décès du fœtus. Le mécanisme de formation de ces hématomes n'est pas encore élucidé. Le premier cas a été rapporté en 1977 par Mc Donald et coll. Le diagnostic est posé le plus souvent par l’échographie. L'imagerie par résonance magnétique peut être réalisée si existe un doute diagnostique. L'hématome est le plus souvent bilatéral, souvent associé à une hydrocéphalie. Les causes habituelles d'hémorragies (traumatismes, coagulopathies, anomalies vasculaires, états infectieux) sont rarement trouvées, contrairement à l'hématome sous-dural du nouveau-né, presque exclusivement post-traumatique. Le pronostic est souvent mauvais en 1992, sur les 8 cas décrits dans la littérature ; ils retrouvent 37,5% de décès et 62,5% de séquelles neurologiques. Nous rapportons l'observation d'une patiente de 24ans, troisième geste primipare, admise à la consultation d’échographie pour une malformation cérébrale à 28 semaines d'aménorrhée(SA). Ceci a révélé une grossesse mono fœtale évolutive en présentation de siège dont la biométrie correspondant l’âge gestationnel avec une image ovalaire finement échogène prenant tout le pourtour du crâne et exerçant un effet de masse sur les différentes structures cérébrales. A 32SA, le fœtus a décédé et la patiente a accouché par voie basse.

**Figure 1:**
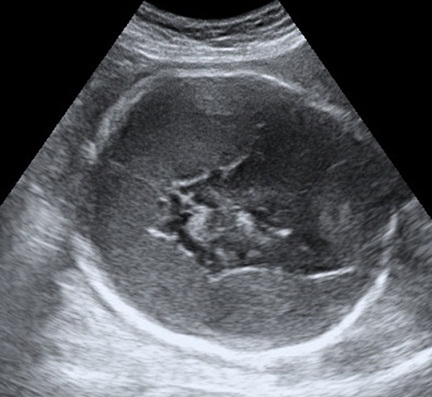
Image finement échogène prenant tout le pourtour du crâne et exerçant un effet de masse sur les différentes structures du parenchyme cérébrale

